# First molecular evidence of *Clostridium perfringens* in adult *Fasciola spp*. isolates in cattle hosts

**DOI:** 10.3389/fvets.2022.967045

**Published:** 2022-09-02

**Authors:** Burcu Karagulle, Figen Celik, Sami Simsek, Haroon Ahmed, Yujuan Shen, Jianping Cao

**Affiliations:** ^1^Department of Microbiology, Faculty of Veterinary Medicine, University of Firat, Elazig, Turkey; ^2^Department of Parasitology, Faculty of Veterinary Medicine, University of Firat, Elazig, Turkey; ^3^Department of Biosciences, COMSATS University Islamabad (CUI), Islamabad, Pakistan; ^4^National Institute of Parasitic Diseases, Chinese Center for Disease Control and Prevention, Shanghai, China; ^5^Chinese Center for Tropical Diseases Research, Shanghai, China; ^6^Key Laboratory of Parasite and Vector Biology, National Health Commission of the People's Republic of China, Shanghai, China; ^7^WHO Collaborating Center for Tropical Diseases, Shanghai, China; ^8^The School of Global Health, Chinese Center for Tropical Diseases Research, Shanghai Jiao Tong University School of Medicine, Shanghai, China

**Keywords:** cattle, *Clostridium perfringens*, *Fasciola spp*., liver, necrotic hepatitis

## Abstract

Fasciolosis is a parasitic disease caused by *Fasciola spp*. It is a prevalent helminth infection globally. Clostridial hepatitis is a general name refer to disorders caused by a few clostridial agents that most severely affect the liver. Migration of young parasite forms (mostly *Fasciola hepatica*) in the parenchymal tissue of the liver causes necrosis and anaerobic environment, stimulating the proliferation of *C. novyi* type B spores. This study investigated the occurrence of *Clostridium spp* in adult *Fasciola spp* isolates. Isolates (*n* = 100) were collected from the bile ducts of infected cattle after slaughter. Total genomic DNA was extracted from each sample. A multiplex-PCR based on the flagellin C (*fli*C) gene was used for quick identification of *C. chauvoei, C. haemolyticum, C. novyi types* A and B, and *C. septicum*. In addition, a pair of primers *Cpa* (F) and *Cpa* (R) were used for detection of the *C. perfringens* alpha toxin gene. The products were sequenced. No band was obtained after multiplex-PCR of the *fliC* gene. A 247 bp band was detected in two isolates using the *Cpa* primers. BLAST analysis of these two isolates characterized both as *C. perfringens* alpha toxin. This is the first description of the molecular detection of *C. perfringens* in flukes. Further studies are needed to investigate whether Clostridum species is also carried by other developmental forms (egg and larval stages) of *Fasciola spp*.

## Introduction

*Fasciola hepatica* (liver fluke) is a common, globally prevalent digenetic trematode. The infections caused by liver fluke result in significant economic losses among livestock (1). In small ruminants (sheep and cattle) the disease is mainly caused by *F. hepatica* (2). According the World Health Organization, it is a foodborne trematode infection that has increased significantly in recent years. It has been described on all continents and has affected at least 2.7 million people (3). The pathogenesis of fasciolosis and related clinical findings may vary according to the degree of infectivity and number of the metacercariae, time for the infection to develop, host type, host immunity, and size of the liver (4). *Clostridium* spp. are anaerobic spore-forming bacteria commonly found in soil. However, they also live as commensal microorganisms in the intestinal microbiota of humans and many animals (5, 6). Histotoxic clostridia mainly cause a necrotic infection known as gas gangrene in the subcutaneous tissue and muscles of ruminants, horses, and other domestic and wild animals. The etiological agents of the disease, alone or in combination, are *Clostridium septicum, C. chauvoei, C. novyi* type A, *C. perfringens* type A, and *C. sordellii*. (7–9). *C. novyi* type B is responsible for infectious necrotic hepatitis, also known as black disease, among sheep and cattle (10, 11).

Due to endemic nature, bacillary hemoglobinuria is only prevalent in regions where *F. hepatica* is abundant. Parasites are the likely primary cause of lesions (12–14). Grazing animals ingest *C. novyi* type B spores. Subsequently, the spores are passed from the intestine and migrate to liver by mixing with the phagocytic cells *via* portal circulation. Spores can be latent for several months in the cytoplasm of phagocytic cells (11, 13). Immature parasite forms that cause liver damage in the local anaerobic environment prelude the germination and toxin production of *C. novyi* type B spores. In some regions, the relationship between infectious necrotic hepatitis and fascioliasis may vary depending on the intensity of *F. hepatica* metacercariae in pastures (15).

The present study investigated the occurrence of *Clostridium spp*. in adult *F. hepatica* species collected from cattle.

## Materials and methods

### Sample collection

Adult *Fasciola spp* isolates (*n* = 100) were collected from the bile duct of infected cattle after slaughter at a slaughterhouse in Elazig province, Turkey. Parasites were investigated by making sections from each lobe of the whole livers, the main bile ducts and also a few sections of the liver parenchyma tissue. Only an adult parasite and preferably uncalcified live adult parasites were collected from the bile ducts. Flukes were collected with forceps, taking care to collect them whole, and brought to the laboratory in a carrier container containing saline. All adult flukes were washed with 1 × PBS (pH = 7.4 here and hereafter) at least five times to remove bacteria (16). The washed flukes were stored in 70% ethanol at −20°C. The work has been approved from Ethical Committee of Firat University Turkey under No. 232.

### Genomic DNA (gDNA) isolation

A small piece was acquired from the anterior (front) end of the adult parasite, placed on a slide, and cut with a sterile blade. The piece was washed at least five times by 1 × PBS to remove ethanol. The gDNA from each sample was extracted by a commercial kit (DNA isolation kit from tissue and cell culture, Hibrigen Biotechnology, Turkey, Lot: 0222-OY-2033) as described by the manufacturer's instructions. All isolated gDNAs were measured by NanoDrop (Cat no: ND-2000, Thermo Fisher Scientific) and re-isolated from the samples obtained under 10 ng/μl of gDNA. The extracted gDNA was kept at −20°C until used.

### PCR amplification

A multiplex-PCR (17) based on the flagellin C (*fli*C) gene was used to rapidly identify *C. chauvoei, C. haemolyticum, C. novyi* types A and B, and *C. septicum* which was the primer designations and sequences are summarized in Table 1. The PCR cycles were started with pre-denaturation at 94°C during 5 min, then 30 cycles denaturation (1 min/94°C), annealing (1 min/55°C), and extension (90 sec/72°C) were performed. The last extension was carried out at 72°C during 7 min. In addition to the multiplex-PCR, a pair of primers *Cpa* (F) 5′-TGCTAATGTTACTGCCGTTGATAG-3′ and *Cpa* (R) 5′-ATAATCCCAATCATCCCAACTATG-3′ were used for detection of the *C. perfringens* alpha toxin gene (18). 5 μl of 10 × PCR buffer (GeneDirex, Taiwan), 200 μM of each dNTP's (GeneDirex, Taiwan), 20 pmol of each primer, 0.2 μl of TaqDNA polymerase (1.25 IU) (GeneDirex), 32.8 μl sterile distilled water and 5 μl of gDNA were added to PCR mixture. Amplification was carried out for 5 min at 94°C, followed by 30 cycles of 60 s at 94°C, 60 s at 56°C, and 2 min at 72°C, with a further extension step of 10 min at 72°C. PCR was performed using a thermal cycler (SensoQuest GmbH, Germany). *Clostridium perfringens* ATCC-3624 Type A strain's DNA was used as positive control. As a negative control 5 μl distilled water was used in each PCR. The PCR products were examined by 1.4% agarose gel electrophoresis. Under ultraviolet (UV) illumination, the amplified bands were excised from the gel and were subsequently purified by a PCR gel purification kit (Hibrigen, Turkey). The isolated products were used in a forward primer-based unidirectional sequence analysis.

**Table 1:** Primers used for PCR amplification of *Clostridium* species.

**Primer names**	**Sequence**	**Position**
Bacterial strain primer		
FlaF	5′-AGAATAAACAGAGCTGGAGATG-3′	135–156
***C. novyi*** **type A strain**		
FlanaR	5′-CGCCTACTTGGAAAGTTACTC-3′	472–452
***C. novyi*** **type B strain**		
FlanbR	5′-TTATGCTAACTTTAGCTGCGTC-3′	551-530
***C. septicum*** **strain**		
FlaseR	5′-TTTATTGAATTGTGTTTGTGAAG-3′	421–399
***C. haemolyticum*** **strain**		
FlahaR	5′-CTGCTGTACCTTCTATGAACC-3′	819–799
***C. chauvoei*** **strain**		
FlachR	5′-TACTAGCAGCATCAAATGTACC-3′	669–648

### Phylogenetic analyses

FinchTV 1.4.0 (Geospiza Inc., USA; http://www.geospiza.com) was used to view the sequence data. The sequence ends were cut from appropriate points by comparing published sequences using the BLAST search (http://www.ncbi.nlm.nih.gov/BLAST). Nucleotide alignments were performed by using CLC Sequence Viewer 8 (19). The sequences were uploaded to MEGA X (19) and ClustalW aligned for phylogenetic analysis. The most suitable phylogenetic tree model for the sequences was the Tamura 3-parameter model and Has Invariant Sites (T92+I). The phylogenetic tree was generated by bootstrap testing (1,000 replicates) by the Maximum Likehood method (20).

## Results

**Figure 1:**
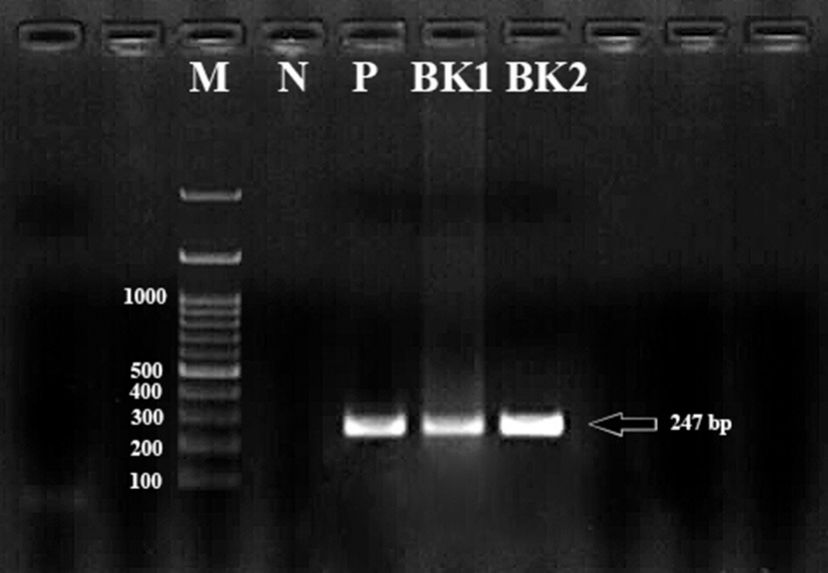
The bands formed as a result of PCR amplification of the *Cpa* (alpha toxin encoding gene) gene fragment of *Clostridium perfringens* cattle isolates corresponding to approximately 247 bp in size. M, Marker (100 bp); P, Positive control; N, Negative control; BK1 and BK2, *Clostridium perfringens* alpha toxin encoding gene.

Isolation of gDNA was successful in all 100 adult parasites. No band was obtained by multiplex-PCR of the *fliC* gene for the rapid identification of *C. chauvoei, C. haemolyticum, C. novyi* types A and B, and *C. septicum*. After PCR amplification of all the gDNA, a 247 bp band was detected in two isolates (BK1, BK2) using the *Cpa* primers (Figure 1). BLAST analysis of BK1 and BK2 characterized as *C. perfringens* alpha toxin producers.

**Figure 2:**
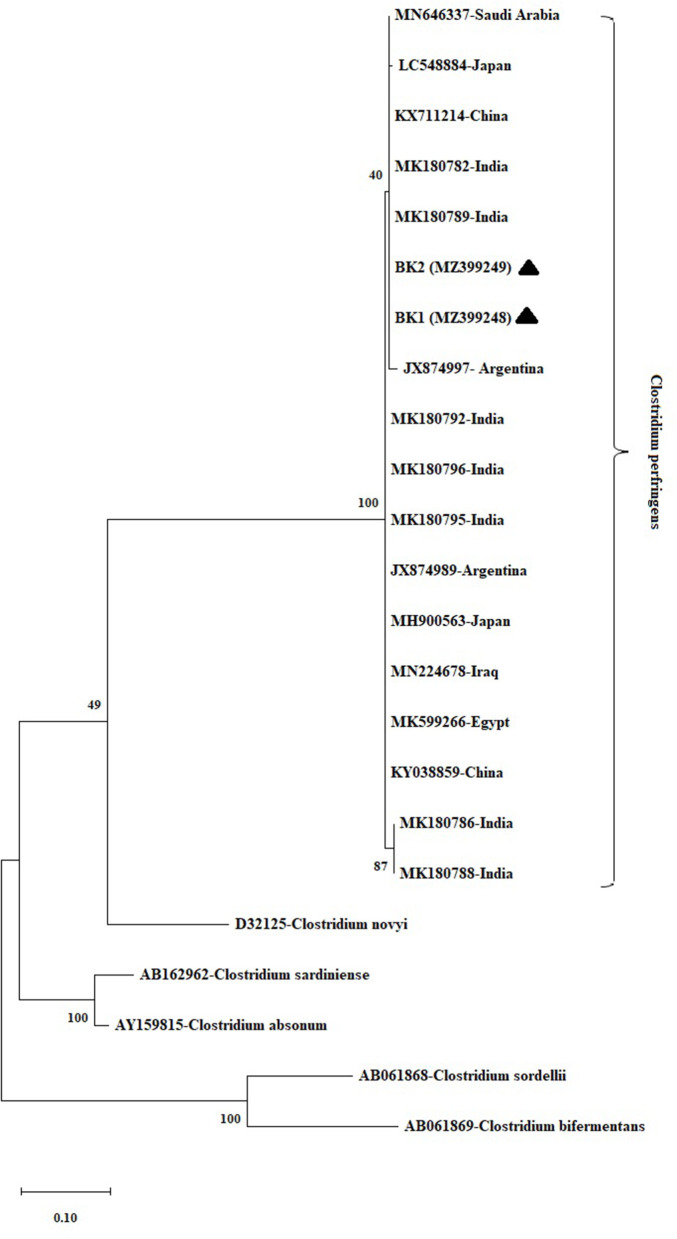
Phylogenetic tree of *Clostridium perfringens* samples (*n* = 2) detected in adult Fasciola spp, based on a 210 bp long fragment of the Cpa gene. *C. sardiniense* (AB162962), *C. novyi* (D32125), *C. absonum* (AY159815), *C. sordellii* (AB061868), and *C. bifermentans* (AB061869) were used as outgroup sequences for phylogenetic tree construction. MEGA X was used to construct a Maximum Likelihood tree based on the T92+I model. The reliability of the tree was assessed by 1,000 bootstrap replications. *Fasciola spp*. samples (MZ399248, BK1; MZ399249, BK2) are indicated by black triangles.

The obtained sequences were submitted in the GenBank database (Accession No. MZ399248 and MZ399249). Phylogenetic tree of *C. perfringens* (*Fasciola spp* samples from Turkey, *n* = 2) based on a trimmed 210 bp fragment of the alpha toxin gene is shown in Figure 2. *C. perfringens* (MK180786, MK180788, MK180789, MK180792, MK180796, MK180782, MK180795, KX711214, JX874989, JX874997, MN646337, LC548884, MH900563, MN224678, MK599266, and KY038859) were used as reference sequences from different isolation sources and different geographical regions. *C. sardiniense* (AB162962), *C. novyi* (D32125), *C. absonum* (AY159815), *C. sordellii* (AB061868), and *C. bifermentans* (AB061869) were used as outgroup sequences for phylogenetic tree construction.

## Discussion

Infections caused by the *F. hepatica* trematode parasite were first described in the 13^th^ century. Fasciolosis continues to be a problem globally, with appreciable economic losses in humans and animals. During the hepatic phase of *F. hepatica*, after 6–7 weeks of passage through the hepatic cells, it is believed that immature flukes enter the bile ducts of definitive hosts and develop sexually (21).

The *C. novyi* type B strain invades the liver and produces highly potent exotoxins, causing black disease (infectious necrotic hepatitis). Disease occurs in non-immune animals when exotoxins are produced by *C. novyi* in the anaerobic environment of the liver. The micro- and-macroenvironmental niches allow germination of *C. novyi* spores and are mostly due to the migration of liver flukes. *C. novyi* type B spores are present in the soil and feces of domesticated animals. The spores resist harsh climatic conditions (22). The occurrence of *Clostridium spp*. in the liver has been described however in these reports (12, 22, 23), the bacteria were isolated from liver tissue. Although not proved, *Fasciola spp* have been mentioned to be possible predisposing factors for infectious necrotic hepatitis (24). Therefore, it can be said that farms contaminated with any of these parasites are at risk of occuring infectious necrotic hepatitis (11). However, no record has been found regarding the presence of this bacterium in immature or adult liver flukes.

There is no previously reported *C. perfringens* sequence from Turkey. Therefore, our sequences could not be compared with Turkish isolates. As seen in Figure 2, it has been determined that our sequences (MZ399248 and MZ399249) showed close similarities with the sequences reported from other countries of the world.

Grazing animals can ingest spores of *Clostridium spp*. The spores are absorbed in the gut where they are phagocytized by local macrophages. Hepatic, spleen, and stem cell involvement may result. They remain latent in the cytoplasm for several months. Type B spores have been detected in sheep liver within 24 h after the oral administration under experimental conditions (12). In endemic regions, latent infections occur in the liver of many healthy hosts (including cattle, sheep, and dogs). Black disease is a serious infection of sheep. The most frequent cause of emergence is the transmission of common liver fluke larvae *F. hepatica*. This disease is responsible for causing rapid deaths among sheep without any warning signs. Typical and diagnostic lesions occur in the liver. The liver is a location of acute traumatic hemorrhagic lesions associated with acute fascioliasis and/or chronic cholangiohepatitis. Coagulation necrosis occurs in a confined tunnel zone caused by flukes (25).

## Conclusion

This is the first study describing the molecular detection of *C. perfringens* in flukes. The available evidence suggests that young flukes harboring Clostridium may transport the bacteria from the intestinal tract to the liver. This study shows us that flukes should also be taken into account in the spread of *C. perfringens*. In addition, it can be said that clostridial agents should be considered in the pathogenesis of liver fluke infections.

## Data availability statement

The original contributions presented in the study are included in the article/supplementary material, further inquiries can be directed to the corresponding author/s.

## Ethics statement

The animal study was reviewed and approved by the work has been approved from Ethical Committee of Firat University Turkey under No. 232.

## Author contributions

BK, FC, JC, and SS conceived the study, performed the experiments, analyzed and curated the data, and supervised the study and revision of the manuscript. HA and SS participated in the methodology, formal analysis, data curation, and contributed their scientific advice. BK, FC, SS, YS, JC, and HA drafted the manuscript and prepared the manuscript for publication and revision. All authors have read and agreed to the published version of the manuscript.

## Funding

This study was supported by the National Natural Science Foundation of China (Nos. 81971969 and 81772225 to JC) and the Fifth Round of Three-Year Public Health Action Plan of Shanghai (No. GWV-10.1-XK13 to JC). The funders had no role in the study design, the data collection and analysis, the decision to publish, or the preparation of the manuscript.

## Conflict of interest

The authors declare that the research was conducted in the absence of any commercial or financial relationships that could be construed as a potential conflict of interest.

## Publisher's note

All claims expressed in this article are solely those of the authors and do not necessarily represent those of their affiliated organizations, or those of the publisher, the editors and the reviewers. Any product that may be evaluated in this article, or claim that may be made by its manufacturer, is not guaranteed or endorsed by the publisher.
